# A Toxicological Evaluation of Germanium Sesquioxide (Organic Germanium)

**DOI:** 10.1155/2020/6275625

**Published:** 2020-04-04

**Authors:** Robin A. Reddeman, Róbert Glávits, John R. Endres, Timothy S. Murbach, Gábor Hirka, Adél Vértesi, Erzsébet Béres, Ilona Pasics Szakonyiné

**Affiliations:** ^1^AIBMR Life Sciences, Inc., 2800 E. Madison St., Suite 202, Seattle, WA 98373, USA; ^2^Toxi-Coop Zrt., H-1122 Budapest, Magyar Jakobinusok Tere 4/B, Hungary

## Abstract

A battery of OECD- and GLP-compliant toxicological studies was performed to assess the safety of a highly purified germanium sesquioxide, an organic form of the naturally occurring, nonessential trace element germanium. Germanium dioxide and germanium lactate citrate (inorganic germaniums) have been shown to induce renal toxicity, whereas germanium sesquioxide (an organic germanium) has been shown to have a more favorable safety profile. However, past toxicity studies on germanium sesquioxide compounds have not clearly stated the purity of the tested compounds. In the studies reported herein, there was no evidence of mutagenicity in a bacterial reverse mutation test or an in vitro mammalian chromosomal aberration test. There was no genotoxic activity observed in an in vivo mammalian micronucleus test at concentrations up to the limit dose of 2000 mg/kg bw/day. In a 90-day repeated-dose oral toxicity study in Han:WIST rats conducted at doses of 0, 500, 1000, and 2000 mg/kg bw/day by gavage, there were no mortalities, treatment-related adverse effects, or target organs identified. The no-observed-adverse-effect-level (NOAEL) was determined to be 2000 mg/kg bw/day.

## 1. Introduction

Germanium is a naturally occurring, nonessential trace element found in soil, rocks, fresh water, plants (e.g., ginseng, aloe, and garlic), and in most foods, albeit in trace amounts [1]. Use of germanium-containing compounds as dietary supplements and medicines for promoting health became popular in the 1970s and 80s in Japan, Great Britain, and elsewhere; however, shortly thereafter, case reports of toxic effects after chronic consumption of germanium-containing preparations began to appear [1–7]. Adding to the concern, the errors of reporting with regard to which specific germanium compound(s) was(ere) consumed caused much confusion regarding the overall safety of germanium [2].

Subsequent toxicological investigations into germanium-containing compounds revealed significant differences in toxicity profiles between germanium dioxide, (inorganic germanium) and germanium sesquioxide (Ge-132, an organic germanium) [1, 2, 8]. While germanium dioxide has been shown to induce renal toxicity in animal models [9, 10] and has been identified as one of the toxic agents in human case reports of renal toxicity (as has germanium lactate citrate) [1, 3–7, 11], Ge-132 has been shown to have a more favorable safety profile [8, 12, 13]. Even so, the toxicological and clinical data that has been published on Ge-132 is limited. A 6-month repeated-dose oral toxicity study on Ge-132 (1000 mg/kg body weight (bw), 5 days per week, ∼714 mg/kg bw/day) in rats was published in 1992 and indicated some adverse kidney effects [13]; however, the purity of the test item was not provided and only a single dose group was utilized. In a 24-week-long oral study with a 16-week-long recovery period comparing the effects of germanium dioxide (75 mg/kg bw/day) to Ge-132 (120 mg/kg bw/day), germanium dioxide was found to have significant systemic and renal toxicity that persisted for 16 weeks after discontinuation of treatment [8]. In the animals administered Ge-132, blood urea nitrogen levels fluctuated; however, neither systemic toxicity nor renal abnormalities were observed. In another 6-month oral toxicity study, Ge-132 was found to induce no toxic effects and no abnormalities at doses of 30, 300, and 3000 mg/kg bw/day but the test item purity and details of the study methods and results were not provided [12]. In a 26-week carcinogenicity study, Ge-132 (≥99% purity) added to the diet of mice at concentrations of 0.3, 0.8, and 2.5% did not cause any treatment-related increase in neoplasms as compared to the control [14].

The lack of conclusive data makes it difficult to rule out the possibility of effects from chronic ingestion, or ingestion at high doses, of Ge-132. Further studies into the safety profile of Ge-132 are of particular interest because of its potential use as a food ingredient or supplement for the purposes of supporting human health or providing therapeutic benefit [13, 15]. Thus, we submit herein an in vitro and in vivo toxicological assessment of a highly pure Ge-132.

## 2. Materials and Methods

All main tests were conducted in accordance with the Organization of Economic Cooperation and Development (OECD) Principles of Good Laboratory Practices (GLP), ENV/MC/CHEM (98)17 [16]. The studies use methods as previously described by Clewell et al. [17], Marx et al. [18], and Reddeman et al. [19] and the methods section partly reproduces our wording. All chemical reagents, solvents, pharmaceuticals, and other chemicals used in the studies were of analytical or pharmaceutical grade.

### 2.1. Test Item

**Figure 1:**
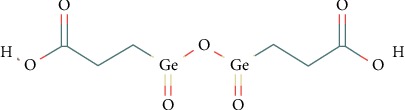
Structural formula of Ge-132 [20].

The test item, Ge-132, (bis(2-carboxyethyl)germanium sesquioxide, CAS registry number: 12758-40-6, molecular weight 339.42, molecular formula C_6_H_10_Ge_2_O_7,_ see structural formula in Figure 1) is an organic form of the naturally occurring, nonessential trace element germanium and was manufactured and supplied by the sponsor, Designed Nutritional Products (Orem, Utah). Synthesis of Ge-132 begins with chemical transformation of germanium dioxide (GeO_2_) followed by pH adjustment, filtration, washing, and drying to produce a ≥99.6%pure white crystalline powder that contains between 42.5–43.1% elemental germanium and <50 ppm GeO_2_ impurity. In order to ensure the production of a finished product meeting the high purity specification, following dissolution of GeO_2_, mechanical means are employed to remove any trace amounts of undissolved GeO_2_ followed by the addition of a stoichiometric excess of acrylic acid to ensure completion of the nonreversible reaction, which is verified by interim analysis before proceeding to the next step. Purity and GeO_2_ impurity are confirmed on each batch of Ge-132, with a GeO_2_ limit of detection of ∼0.5 ppm, using high-performance liquid chromatography coupled with inductively coupled plasma mass spectrometry based on independently validated modifications to the methods of Krystek et al. [21, 22]. Positive identification and suitable chemical purity of the test item (lot 4845 for genotoxicity tests and 4895 for the 90-day study) were verified by the lab based on the sponsor-provided analytical certificate.

### 2.2. Test Methods: In Vitro Tests

#### 2.2.1. Bacterial Reverse Mutation Test

The study was performed following procedures established by Ames et al. [23], Maron and Ames [24], Kier et al. [25], and Venitt and Parry [26] and according to the following: OECD test guideline 471 [27], and the laboratory's SOP and utilized *Salmonella typhimurium* strains TA98, TA100, TA1535, and TA1537 and *Escherichia coli* strain WP2 *uvrA* (Moltox, Inc.) in the presence and absence of a metabolic activation system (S9-mix; rat liver S9 fraction from Moltox, Inc., USA).

Based on the preliminary solubility and concentration range finding test results, the main (plate incorporation procedure) and confirmatory (preincubation procedure) tests were conducted using ultrapure water (ASTM Type I) as the vehicle for the test item, sodium azide (SAZ, Merck KGaA, Germany, Darmstadt), and methyl-methanesulfonate (MMS, Sigma-Aldrich Co., Germany, USA), and dimethyl sulfoxide (DMSO, Merck KGaA, Germany, Darmstadt) was the vehicle for 4-nitro-1,2-phenylene-diamine (NPD, Merck KGaA, Germany, Darmstadt), 9-aminoacridine (9AA, Merck KGaA), and 2-aminoanthracene (2AA, Sigma-Aldrich, Germany, USA). Test item concentrations were 5000, 1600, 500, 160, 50, and 16 *μ*g/plate. Due to the solubility of the test item, a treatment volume of 0.25 mL was used. The higher than usual volume did not significantly dilute the top agar and did not change its composition; parallel investigation of vehicle controls proved the acceptability of the higher treatment volume. Positive and negative controls were chosen according to the cited guidelines and literature. The sensitivity, reliability, and promutagen activation potential of the metabolic activation system was certified by the supplier using known controls and further investigated with positive control solutions. The test solution was freshly prepared at the beginning of each experiment (slight heat was applied to improve the dissolution of the test item).

Manual counting determined the colony numbers, and from this, the mean values, standard deviations, and mutation rates were calculated. According to the test guidelines and established criteria of the laboratory, the test item was considered mutagenic if the following applies:

- A concentration-related increase in revertant colonies occurred and/or;
- A reproducible biologically relevant positive response for at least one dose group occurred in at least one strain with or without metabolic activation.

An increase was considered biologically relevant if the following applies:

- The number of reversions in strains *S. typhimurium* TA98 and/or TA100 and/or *E. coli* WP2 *uvrA* was at least twice as high as the number of reversions of the vehicle control (reversion rate ≥ 2) and/or;
- The number of reversions in strains *S. typhimurium* TA1535 and/or TA1537 was at least three times higher than the number of reversions in the vehicle control (reversion rate ≥ 3).

The test item was considered nonmutagenic if the criteria for a mutagenic response were not observed. Because biological relevance was the criterion applied for the interpretation of results, no statistical evaluation was conducted.

#### 2.2.2. In Vitro Mammalian Chromosomal Aberration Test

The test was performed in compliance with internationally accepted guideline OCED 473 [28] in cultured Chinese Hamster lung V79 cells (European Collection of Cell Cultures).

Based on the results of the preliminary solubility and cytotoxicity tests, Dulbecco's modified Eagle's medium (DME, Sigma-Aldrich, Germany, USA) was selected as the vehicle for preparation of the test item solutions and test item concentrations were chosen for the main test. The positive control for use without metabolic activation was ethyl methanesulfonate (EMS, Sigma-Aldrich Co., Germany, USA) dissolved in DME to final concentrations of 0.4 or 1.0 *μ*L/mL as it is a known mutagen and clastogen, and the test facility has a broad historical database documenting its use. The positive control with metabolic activation was cyclophosphamide monohydrate (Sigma-Aldrich, Germany, USA) dissolved in DME to achieve the final concentration of 5.0 *μ*g/mL.

In Experiment A, duplicate V79 cultures (5 × 10^5^ cells/group) were exposed to the respective vehicle control, positive control, and each test item concentration (500 *μ*g/mL, 1000 *μ*g/mL, 2000 *μ*g/mL) with and without metabolic activation for a period of 3 hours. After the exposure period, the cells were washed with DME and sampling was taken 20 hours from the start of treatment.

Experiment B was performed as described for Experiment A except for the exposure period without metabolic activation was 20 hours with sampling taken 20 and 28 hours after treatment initiation. The exposure period with metabolic activation remained 3 hours, but sampling was taken 28 hours after initiation of treatment.

Per test guidelines, cell cultures were treated with colchicine (0.2 *μ*L/mL, Sigma-Aldrich Co., Germany, USA) 2.5 hours before sampling and then slides were prepared and scored blind. The nomenclature and classification of chromosome aberrations were based upon ISCN and Savage [29–31]. For statistical analysis, a chi-square test and regression analysis were performed.

### 2.3. Animal Studies

The Institutional Animal Care and Use Committee of Toxi-Coop Zrt. permitted the conduct of these animal studies which were conducted according to the National Research Council Guide for the Care and Use of Laboratory Animals and in compliance with the principles of the Hungarian Act 2011 CLVIII (modification of Hungarian Act 1998 XXVIII) and Government Decree 40/2013 regulating animal protection.

#### 2.3.1. In Vivo Mouse Micronucleus Test

The study was performed in compliance with OECD 474 [32]. Specific pathogen-free CRL:NMRI BR mice, aged eight weeks and with body weights of 34.3–37.9 g, were utilized for this study. Acclimatization and husbandry of the animals were carried out in accordance with the cited test guidelines.

Aqueous methylcellulose (1%, Molar Chemicals Kft., Hungary) was used as the vehicle control and solvent for the test item. Cyclophosphamide dissolved in sterile water (Naturland Kft, Hungary) was used for the positive control. The test item formulations were prepared fresh each day of dosing to achieve concentrations of 50 mg/mL, 100 mg/mL, and 200 mg/mL and were used within two hours.

On the basis of the results of the GLP preliminary toxicity test, doses of 500, 1,000, and 2,000 mg/kg/bw were selected for the main test for administration of two doses by gavage, 24 hours apart. Male CRL:NMRI BR mice were randomly divided into five groups: a vehicle control, positive control, and the three test groups. The positive control, cyclophosphamide 60 mg/kg/bw, was administered once intraperitoneally. The mice were examined for visible signs of reactions to treatment immediately after dosing and at intervals until sacrifice (by cervical dislocation). In the test item and solvent control groups, the sampling was made once 24 hours after the second treatment. In the positive control group, sampling was performed 24 hours after the beginning of treatment. Bone marrow was obtained from two exposed femurs of mice from every time point immediately after sacrificing and smears were prepared on standard microscopic slides and evaluated per test guidelines for the incidence of micronucleated cells and the proportion of immature among total (immature + mature) erythrocytes. Statistical analysis was performed using the Kruskall Wallis Nonparametric one-way analysis of variance (ANOVA) test and regression analysis.

#### 2.3.2. 90-Day Repeated-Dose Oral Toxicity Studies in Rats

The study followed procedures and conducted examinations as described by OECD test guideline 408 (as a minimum standard) [33].

SPF Han:WIST rats (Toxi-coop, Zrt, Budapest, Hungary) 52–66 days old weighing 212–261 g (males) and 143–168 g (females) were acclimatized and housed under environmental conditions in accordance with OECD guideline 408. Animals received food (ssniff®SM R/M-Z + H complete diet for rats and mice produced by ssniff Spezialdiäten GmbH, Soest, Germany) and potable tap water ad libitum except for overnight food deprivation before blood sampling.

Dose selection was based on data from an unpublished OECD 407 compliant 14-day repeated-dose oral toxicity study in Han:WIST rats in which no mortalities or toxic effects were observed in any of the examined study parameters and a NOAEL of 2000 mg/kg bw/day was determined. Thus, the test item for the 90-day study was formulated in 1% aqueous methylcellulose to concentrations of 50, 100, and 200 mg/mL for gavage administration of 0 (vehicle only), 500, 1000, and 2000 mg/kg bw/day (10 animals/sex/group) at a 10 mL/kg bw dose volume. In a deviation from GLP, in lieu of analytical control, each formulation was prepared daily just prior to administration and used within four hours after preparation.

Per guidelines, daily and weekly clinical observations were performed and in the last exposure week, using a modified Irwin test [34], a functional observation battery (FOB) was conducted.

Individual body weights were measured, and body weight gain was calculated. Food consumption was determined, and feed efficiency was calculated weekly to coincide with body weight measurements. Ophthalmological examination was performed on all rats during the acclimation period and all animals of the control and high-dose groups prior to test termination.

After an overnight fast, following termination of treatment, blood samples for clinical pathology (hematology and clinical chemistry) were collected from the retroorbital venous plexus under Isofluran CP® anesthesia (Medicus Partner, Kft., Hungary). Gross pathological examinations were performed on every animal following sacrifice by exsanguination from the abdominal aorta after verification of narcosis.

All animals were weighed prior to sacrifice in order to calculate relative organ weights. Full histological examinations were performed on preserved organs and tissues of the control and high-dose group animals and on the basis of macroscopic findings in low- and mid-dose group animals.

Male and female rats were evaluated separately, and statistical analyses were performed. Bartlett's homogeneity of variance test was used to assess the heterogeneity of variance between groups; if no significant heterogeneity was detected, then an ANOVA was carried out. In the case of a positive result, Duncan's Multiple Range test was used to assess the significance of intergroup differences. Where significant heterogeneity was found, the normal distribution of data was examined by the Kolmogorov-Smirnov test. In the case of a nonnormal distribution, the nonparametric method of Kruskal-Wallis ANOVA was used. If there was a positive result, the intergroup comparisons were performed using the Mann–Whitney U-test. Statistical significance was assigned when the *p* value was <0.05. Frequencies of clinical signs, ophthalmoscopy, pathological, and histopathological findings by sex and dose were calculated but not subjected to statistical analysis.

## 3. Results and Discussion

### 3.1. Bacterial Reverse Mutation Test

In the confirmatory test, the revertant colony numbers for *E. coli* WP2 uvrA at 5,000 *μ*g/plate with metabolic activation were above the range of the corresponding vehicle control historical data. This increase was not dose-related and the mutation rate (1.67) was well below the genotoxicological threshold for a positive test. No background inhibition and no concentration-related or biologically relevant increases in revertant colony numbers of any of the five tester strains were observed, at any concentration level, either in the presence or absence of the metabolic activation in the initial or confirmatory mutation tests (see Tables 1 and 2). All validity and acceptability criteria of the tests were fulfilled, and all the results were unequivocally negative.

### 3.2. In Vitro Mammalian Chromosomal Aberration Test

The number of aberrations found in vehicle controls was within the range of the historical control data. The concurrent positive controls caused the expected biologically relevant increases in cells with structural chromosomal aberrations as compared to vehicle controls and increases were comparable to the historical control data.

Under the conditions of experiments A and B, Ge-132 did not induce a statistically significant increase in the number of cells with aberrations without gaps at any examined concentration compared to vehicle and historical controls nor were any polyploid or endoreduplicated metaphases found. There were no statistically significant differences between the test item and vehicle control groups and no dose-response relationships were noted (see Table 3). All validity and acceptability criteria of the conducted tests were fulfilled, and all results were unequivocally negative.

### 3.3. In Vivo Mouse Micronucleus Test

No mortality or sex specific effects were observed in the preliminary toxicity test. In the main study, no mortality occurred, and no adverse reactions to treatment were observed in mice of the vehicle and positive controls or in any of the test item groups.

Cyclophosphamide-treated mice showed the expected large, statistically significant increases in the micronucleated polychromatic erythrocyte (MPCE) numbers compared to the vehicle and historical controls, which demonstrated acceptable sensitivity to the test. No biologically or statistically significant increases were observed in the frequency of MPCEs in any test group 24 hours after the second treatment compared to the concurrent vehicle and historical controls. The proportion of PCEs among total erythrocytes at 24 hours sampling time in the 500 and 1000 mg/kg/bw dose groups was similar to that of the vehicle control. The proportion of PCEs among total erythrocytes was slightly, but not statistically or biologically significantly decreased in the 2000 mg/kg/bw group compared to vehicle control, thus, demonstrating exposure of the bone marrow to the test item (see Table 4). All validity and acceptability criteria of the conducted tests were fulfilled, and all results were unequivocally negative.

### 3.4. 90-Day Repeated-Dose Oral Toxicity Study in Rats

There were no mortalities in any group during the 90-/91-day study period (male/female, respectively). In daily clinical observations, paler than normal stools were observed in male and female animals in the 1000 and 2000 mg/kg bw/day groups and softer than normal stools in male and female animals in the 2000 mg/kg bw/day group. While this was likely associated with the test item or its metabolites, possibly the result of an osmotic effect from the increased dosage of the test item, the stool changes were not considered biologically or toxicologically relevant due to a lack of correlating clinical or pathological changes. Transiently decreased activity in one male from the 2000 mg/kg bw/day group was observed between days 51 and 54. Alopecia was observed on the forelimbs of one male and on the abdomen of one female in the control group and on the forelimbs of one male animal from the 500 mg/kg bw/day group from day 85 until the end of the study. Alopecia and decreased activity were not considered test item-related due to their transient and isolated occurrence and/or greater occurrence in control animals. In the detailed weekly clinical examinations, the behavior and physical condition of all treated animals were normal during the entire study period (with the exception of the alopecia described above). There were no differences with respect to the controls in behaviors or reactions to stimuli during the FOB in any treatment group. There were no changes observed on ophthalmologic examination in the 2000 mg/kg bw/day or control groups.

In male rats, there were no statistically significant changes in body weight relative to controls in the low- and mid-dose groups (see Table 5), but body weight gain was significantly affected in all treatment groups at different points during the observation period (see Table 6). The statistically significant decrease in mean body weight gain in high-dose males resulted in lower mean body weight overall. However, the significant differences in mean body weights with respect to control were <10% and, therefore, considered to be without toxicological relevance. The other statistically significant increases and decreases in body weight gain in low- and mid-dose males were sporadic and did not affect overall body weight gain or mean body weights. In females, there were no statistically significant changes in body weight relative to controls (see Supplemental Table 1). High-dose group females demonstrated statistically significant changes in body weight gain, with increases observed between days 77–84 and decreases observed between days 84–89 (see Supplemental Table 2); however, cumulative body weight gain was similar to controls. The changes in body weight gain in females were minor and sporadic and did not affect overall body weight development. Thus, none of these changes (in males and females) were considered toxicologically relevant.

Mean daily food consumption was similar to controls in all dose groups throughout the study (see Supplemental Table 3). Transient and cumulative statistically significant changes were observed for slightly increased feed efficiency values in the 1000 and 2000 mg/kg bw/day males (see Table 7). These changes represent a slightly lower feed efficiency in mid- and high-dose males and were likely due to the lower body weight gain described above. With the exception of an isolated (Week 3 only) improvement in feed efficiency for the low- and high-dose groups, feed efficiency in females was not affected. Therefore, these changes were not considered toxicologically relevant (see Supplemental Table 4).

Several statistically significant changes in hematology parameters were observed among the sexes in the treatment groups (see Table 8, Supplemental Table 5). There were multiple statistically significant changes in the clinical chemistry parameters in the treatment groups (see Table 9). These changes were not considered related to the test item as many were sporadic and within or marginal to the historical ranges while a few were likely artifacts of control values outside the normal range. Moreover, there were no related histological findings. Therefore, these changes were considered to be of little or no biological significance.

Macroscopic examination revealed some sporadic changes in organs of the control and/or treatment groups of both sexes with similar frequencies and/or without dose relation (see Table 10). The observed macroscopic findings were considered incidental, of the nature commonly observed in this strain and age of rat or to have occurred in connection with the exsanguination process (lungs, thymus, and liver) and were not considered test item-related.

Several organ weights (absolute and relative to body weight) in male rats in the mid- and high-dose groups (see Table 11, Supplemental Tables 6–8) were statistically significantly different from controls but organ weights relative to brain weight were comparable to controls. These statistically significant sporadic changes in the 2000 mg/kg bw/day males (absolute and relative to body weight) were likely due to the slightly decreased fasted body weight and were within the limits of normal biological variation as evidenced by their remaining within the normal range of the lab. In females, there were no statistically significant changes in absolute organ weights, except for slightly lower uterine weights in the high-dose group, which were likely due to the number of females in estrous cycle in the control group (6/10 versus 0/10 in the high-dose group). Statistically significant increases in liver weights relative to body and brain weights were observed in low- and high-dose groups; however, they were not dose-related and there were no associated microscopic changes. None of these minor changes, in males or females, were considered test item-related or biologically significant.

With the exception of pyelectasia in several males, microscopic changes were noted in the cecum, epididymides, kidneys, liver, lungs, skin, spleen, and thymus in individual animals and/or with similar frequency in test item and control groups. In females, microscopic changes were noted in the thymus and kidneys of treated animals with similar or greater frequency than controls, and uterine changes occurred with greater frequency in controls (see Table 12). The observed microscopic findings were considered incidental, of the nature commonly observed in this strain and age of rat, present in individual animals and/or were of similar incidence in the control and treated animals; therefore, the changes were not considered test item-related.

The present work contributes further evidence that highly purified and well-characterized Ge-132 has a more favorable toxicity profile than germanium dioxide. As discussed in the introduction, unlike previous works that reported renal toxicity in animal models and human case studies after ingestion of “germanium-containing” compounds, most of which were germanium dioxide-containing, ≥99.6% pure Ge-132 did not cause macroscopic or microscopic renal damage in this 90-day study. Additionally, the pyelectasia observed in the current study was not accompanied by inflammation or degeneration, and hydronephrosis was incidental in one control animal. While there were similar weight and clinical chemistry findings in this and the Anger et al. [13] studies (males only; decreased weight gain, total protein, albumin), none of these findings were considered indicative of renal pathology or of toxicological relevance. The “slight renal dysfunction” reported by Anger et al. after Ge-132 administration of 1000 mg/kg bw/day (5 days per week × 6 months), consisted of slightly, but not statistically significantly, increased creatinine and renal histological findings described as “tubular disease” in some of the treated male animals (“presence of cylinders, swelling of tubulus cells and flocculus deposits”). While it is not clear what the renal findings mean in today's terms, no abnormalities or degenerative renal changes were observed in the current study, nor were kidney function markers adversely affected.

In another study, after oral administration of 120 mg/kg bw/day Ge-132 to female rats, body weight and hematology findings were similar to controls; however, blood urea nitrogen was significantly increased at weeks 4 and 12 followed by a significant decrease at week 20 compared to controls [8]. Serum creatinine and urinalysis results were similar to controls and there were no significant renal histological findings. Miyao et al. reported that no “toxic effects or abnormalities were observed in laboratory, clinical, and pathology examinations,” after 6 months long administration of 30, 300, and 3000 mg/kg bw Ge-132 (no data provided) [12]. In the carcinogenicity study, Doi et al. did not find histopathological renal changes in any dose group. However, loose stools did occur for all animals of the 2.5% dose group (similar to findings for the high-dose group in the current study), and in some animals, dilatation of the cecum was observed. The authors speculated that these dose-related changes might be due to osmotic pressure in the cecum [14]. In all of these studies, as in the current study, Ge-132 was found to have no toxic effect at the tested doses.

In the published case reports, chronic consumption of germanium-containing compounds (consisting mostly of inorganic germanium) resulted in renal failure. There is no evidence of this occurring in the current studies on Ge-132.

## 4. Conclusions

In conclusion, the genetic toxicological studies reported herein provide evidence that Ge-132 of ≥99.6% purity does not exhibit mutagenic, clastogenic, or in vivo genotoxic potential under the applied test systems up to the maximum recommended test concentrations or limit dose, respectively. No mortality or adverse effects were seen, and no target organs were identified in male or female Han:WIST rats after 90 days of oral administration of Ge-132 at doses of 500, 1000, or 2000 mg/kg bw/day. Based on the observations in this 90-day study, the NOAEL was determined to be 2000 mg/kg bw/day.

## Figures and Tables

**Table 1:** Summary of the results of the bacterial reverse mutation test, initial mutation test.

Initial mutation test (plate incorporation test)																				
Concentrations (*μ*g/plate)	*Salmonella typhimurium* tester strains																*Escherichia coli*			
	TA 98				TA 100				TA 1535				TA 1537				WP2 *uvrA*			
	−S9		+S9		−S9		+S9		−S9		+S9		−S9		+S9		−S9		+S9	
Mean values of revertants per plate mutation rate (MR)	Mean	MR	Mean	MR	Mean	MR	Mean	MR	Mean	MR	Mean	MR	Mean	MR	Mean	MR	Mean	MR	Mean	MR

Untreated control	29.3	1.52	26.3	0.91	96.3	1.12	101.3	0.89	8.7	1.04	12.7	1.15	6.3	1.06	7.7	0.96	32.3	0.95	45.3	0.89
DMSO control	**23.7**	**1.00**	**24.7**	**1.00**	*—*	—	**104.3**	**1.00**	—	—	**10.7**	**1.00**	**7.3**	**1.00**	**7.7**	**1.00**	—	—	**47.0**	**1.00**
Ultrapure water control (100 *μ*L)	—	—	—	—	**92.7**	**1.00**	—	—	**9.0**	**1.00**	—	—	—	—	—	—	**35.7**	**1.00**	—	—
Ultrapure water control (250 *μ*L)	19.3	1.00	29.0	1.00	85.7	1.00	113.3	1.00	8.3	1.00	11.0	1.00	6.0	1.00	8.0	1.00	34.0	1.00	51.0	1.00

5000	26.7	1.38	32.0	1.10	91.7	1.07	110.3	0.97	9.7	1.16	13.7	1.24	7.7	1.28	7.0	0.88	54.0	1.59	55.3	1.08
1600	21.7	1.12	30.3	1.05	87.7	1.02	114.0	1.01	13.7	1.64	11.7	1.06	7.3	1.22	6.0	0.75	43.7	1.28	50.0	0.98
500	22.7	1.17	32.3	1.11	91.0	1.06	117.7	1.04	13.0	1.56	12.7	1.15	6.3	1.06	6.3	0.79	33.7	0.99	42.0	0.82
160	25.0	1.29	28.3	0.98	78.7	0.92	119.3	1.05	11.7	1.40	12.0	1.09	7.7	1.28	8.0	1.00	44.7	1.31	45.0	0.88
50	23.7	1.22	31.0	1.07	93.7	1.09	106.7	0.94	11.7	1.40	12.7	1.15	9.7	1.61	8.0	1.00	38.3	1.13	40.3	0.79
16	24.7	1.28	30.3	1.05	98.3	1.15	108.7	0.96	5.3	0.64	12.7	1.15	8.3	1.39	7.3	0.92	39.7	1.17	40.3	0.79

*NPD (4 μg)*	**336.7**	**14.23**	—	—	—	—	—	—	—	—	—	—	—	—	—	—	—	—	—	—
*SAZ (2 μg)*	—	—	—	—	**1173.3**	**12.66**	—	—	**880.0**	**97.78**	—	—	—	—	—	—	—	—	—	—
*9AA (50 μg)*	—	—	—	—	—	—	—	—	—	—	—	—	**399.3**	**54.45**	—	—	—	—	—	—
*MMS (2 μL)*	—	—	—	—	—	—	—	—	—	—	—	—	—	—	—	—	**1058.7**	**29.68**	—	—
*2AA (2 μg)*	—	—	**1634.7**	**66.27**	—	—	**1834.7**	**17.58**	—	—	**256.7**	**24.06**	—	—	**128.3**	**16.74**	—	—	—	—
*2AA (50 μg)*	—	—	—	—	—	—	—	—	—	—	—	—	—	—	—	—	—	—	**235.7**	**5.01**

Abbreviations: 2AA: 2-aminoanthracene; 9AA: 9-Aminoacridine; DMSO, dimethyl sulfoxide; MMS: Methyl-methanesulfonate; MR: Mutation Rate; NPD: 4-Nitro-1,2-phenylenediamine; S-9, metabolic activation; SAZ: sodium azide. ^*∗*^DMSO was the vehicle of the positive control substances: NPD, 9AA and 2AA; ultrapure water was the vehicle of the test item and for SAZ and MMS. The mutation rates of the test item, untreated, and positive controls were calculated using the data from their respective vehicle controls.

**Table 2:** Summary of the bacterial reverse mutation test, confirmatory mutation test.

Confirmatory mutation test (preincubation test)																				
Concentrations (*μ*g/plate)	*Salmonella typhimurium* tester strains																*Escherichia coli*			
	TA 98				TA 100				TA 1535				TA 1537				WP2 uvrA			
	−S9		+S9		−S9		+S9		−S9		+S9		−S9		+S9		−S9		+S9	
Mean values of revertants per plate mutation rate (MR)	Mean	MR	Mean	MR	Mean	MR	Mean	MR	Mean	MR	Mean	MR	Mean	MR	Mean	MR	Mean	MR	Mean	MR

Untreated control	19.0	0.86	32.3	1.01	93.7	1.16	112.0	1.10	9.7	1.00	11.7	0.85	7.7	0.96	7.7	0.70	29.3	0.73	41.7	1.08
*DMSO control*	**21.7**	**1.00**	**30.7**	**1.00**	*—*	*—*	**91.7**	**1.00**	*—*	*—*	**10.3**	**1.00**	**7.3**	**1.00**	**6.7**	**1.00**	*—*	*—*	**39.7**	**1.00**
*Ultrapure water control (100 μL)*	—	*—*	*—*	*—*	**92.0**	**1.00**	*—*	*—*	**9.3**	**1.00**	*—*	*—*	*—*	*—*	*—*	*—*	**30.7**	**1.00**	*—*	*—*
Ultrapure water control (250 *μ*L)	22.0	1.00	32.0	1.00	81.0	1.00	102.0	1.00	9.7	1.00	13.7	1.00	8.0	1.00	11.0	1.00	40.3	1.00	38.7	1.00

5000	27.0	1.23	30.7	0.96	92.7	1.14	104.0	1.02	12.0	1.24	15.3	1.12	6.7	0.83	8.7	0.79	40.3	1.00	64.7	1.67
1600	27.7	1.26	31.0	0.97	86.3	1.07	93.0	0.91	9.3	0.97	14.7	1.07	7.7	0.96	9.7	0.88	32.3	0.80	50.7	1.31
500	29.7	1.35	30.7	0.96	83.7	1.03	94.0	0.92	13.7	1.41	10.3	0.76	7.0	0.88	9.0	0.82	33.7	0.83	49.3	1.28
160	28.0	1.27	19.3	0.60	84.3	1.04	97.7	0.96	10.0	1.03	12.3	0.90	7.0	0.88	10.3	0.94	33.0	0.82	55.0	1.42
50	23.0	1.05	24.3	0.76	86.0	1.06	102.3	1.00	9.3	0.97	14.3	1.05	9.7	1.21	11.3	1.03	33.7	0.83	46.7	1.21
16	21.0	0.95	27.7	0.86	94.7	1.17	104.3	1.02	7.7	0.79	11.3	0.83	8.0	1.00	8.7	0.79	37.7	0.93	41.3	1.07

*NPD (4 μg)*	**386.7**	**17.85**	*—*	*—*	*—*	*—*	*—*	*—*	*—*	*—*	*—*	*—*	*—*	*—*	*—*	*—*	*—*	*—*	*—*	*—*
*SAZ (2 μg)*	*—*	*—*	*—*	*—*	**981.3**	**10.67**	*–*	*–*	**1053.3**	**112.86**	*—*	*—*	*—*	*—*	*—*	*—*	*—*	*—*	*—*	*—*
*9AA (50 μg)*	*—*	*—*	*—*	*—*	*—*	*—*	*—*	*—*	*—*	*—*	*—*	*—*	**570.0**	**77.73**	*—*	*—*	*—*	*—*	*—*	*—*
*MMS (2 μL)*	*—*	*—*	*—*	*—*	*—*	*—*	*—*	*—*	*—*	*—*	*—*	*—*	*—*	*—*	*—*	*—*	**936.0**	**30.52**	*—*	*—*
*2AA (2 μg)*	*—*	*—*	**1018.7**	**33.22**	*—*	*—*	**2154.7**	**23.51**	*—*	*—*	**149.0**	**14.42**	*—*	*—*	**149.7**	**22.45**	*—*	*—*	*—*	*—*
*2AA (50 μg)*	*—*	*—*	*—*	*—*	*—*	*—*	*—*	*—*	*—*	*—*	*—*	*—*	*—*	*—*	*—*	*—*	*—*	*—*	**170.0**	**4.29**

Abbreviations: 2AA: 2-aminoanthracene; 9AA: 9-Aminoacridine; DMSO, dimethyl sulfoxide; MMS: Methyl-methanesulfonate; MR: Mutation Rate; NPD: 4-Nitro-1,2-phenylenediamine; S-9, metabolic activation; SAZ: sodium azide; ^*∗*^DMSO was the vehicle of the positive control substances: NPD, 9AA and 2AA; ultrapure water was the vehicle of the test item and for SAZ and MMS. The mutation rates of the test item, untreated, and positive controls were calculated using the data from their respective vehicle controls.

**Table 3:** Summary of chromosomal aberration test results.

Groups	S9 mix	Treatment time (h)	Harvest time (h)	Mean aberrant cells/150 cells		Number of aberrations	
				Incl. gaps	Excl. gaps	Incl. gaps	Excl. gaps
*Experiment A1*							
Test item							
500 *μ*g/mL	−	3	20	9	4	9	4
1000 *μ*g/mL	−	3	20	8	4	8	4
2000 *μ*g/mL	−	3	20	9	4	9	4
Vehicle control	−	3	20	8	4	8	4
Positive control	−	3	20	39^*∗∗*^	34^*∗∗*^	67^*∗∗*^	43^*∗∗*^
Hist Veh control^4^	−	3	20	4.70–7.82	1.59–4.11	n/a	n/a
Test item							
500 *μ*g/mL	+	3	20	9	4	10	4
1000 *μ*g/mL	+	3	20	9	5	10	6
2000 *μ*g/mL	+	3	20	9	3	9	3
Vehicle control	+	3	20	7	4	8	4
Positive control	+	3	20	51^*∗∗*^	44^*∗∗*^	86^*∗∗*^	63^*∗∗*^
Hist Veh control^4^	+	3	20	4.66–8.12	1.69–4.35	n/a	n/a

*Experiment B* ^*2*^							
Test item							
500 *μ*g/mL	−	20	20	7	3	8	4
1000 *μ*g/mL	−	20	20	8	5	8	5
2000 *μ*g/mL	−	20	20	7	4	7	4
Vehicle control	−	20	20	7	3	7	3
Positive control	−	20	20	45^*∗∗*^	41^*∗∗*^	84^*∗∗*^	58^*∗∗*^
Hist Veh Control^4^	−	20	20	4.44–7.90	1.60–4.27	n/a	n/a

*Experiment B* ^*3*^							
Test item							
500 *μ*g/mL	−	20	28	9	4	9	4
1000 *μ*g/mL	−	20	28	9	3	10	3
2000 *μ*g/mL	−	20	28	8	4	8	4
Vehicle control	−	20	28	8	3	8	3
Positive control	−	20	28	45^*∗∗*^	41^*∗∗*^	78^*∗∗*^	53^*∗∗*^
Hist Veh control^4^	−	20	28	4.31–7.77	1.59–4.11	n/a	n/a
Test item							
500 *μ*g/mL	+	3	28	8	4	9	4
1000 *μ*g/mL	+	3	28	8	4	9	4
2000 *μ*g/mL	+	3	28	9	4	9	4
Vehicle control	+	3	28	9	3	9	3
Positive control	+	3	28	47^*∗∗*^	39^*∗∗*^	70^*∗∗*^	50^*∗∗*^
Hist Veh control^4^	+	3	28	4.96–7.56	1.92–4.12	n/a	n/a

Abbreviations: n/a: not applicable; Veh: vehicle; Hist: historical; incl: including; excl: excluding; ^1^Positive controls: (−S9): Ethyl methanesulfonate (1.0 *μ*L/mL); (+S9): Cyclophosphamide (5.0 *μ*g/mL); ^2^Positive control: (−S9): Ethyl methanesulfonate (0.4 *μ*L/mL); ^3^Positive controls: (−S9) Ethyl methanesulfonate (0.4 *μ*L/mL); (+S9): Cyclophosphamide (5.0 *μ*g/mL); ^4^Numbers reported are the 95% confidence interval. ^*∗*^*p* < 0.05; ^*∗∗*^*p* < 0.01, to the concurrent vehicle control and to the historical vehicle control.

**Table 4:** Summary of mouse micronucleus test results.

Groups mg/kg bw *n* = 5	Sampling time (hours)	Total number of PCE analyzed	PCE/PCE + NCE		MPCE§	
			Mean	±SD	Mean	±SD
Vehicle control^*∗*^^‡^	24	20000	0.53	0.01	5.00	1.00
Test item^‡^						
500	24	20000	0.51	0.01	5.20	1.30
1000	24	20000	0.50	0.01	4.80	0.84
2000	24	20000	0.49	0.01	5.00	1.00
Positive control^*∗*^	24	20000	0.36	0.06	128.60^*∗∗*^	4.56
Historical vehicle control	24	4000	n/a	n/a	4.77	0.94

Abbreviations: MPCE: micronucleated polychromatic erythrocytes; NCE: normochromatic erythrocytes; PCE: polychromatic erythrocytes. ^*∗*^Vehicle control: 1% aqueous methylcellulose; Positive control: 60 mg/kg bw cyclophosphamide. ^*∗∗*^*p* < 0.01. §MPCE per 4000 PCE. ‡dosing occurred twice in 24 hours.

**Table 5:** Summary of body weight (males), 90-day study.

Group^‡^ mg/kg bw/day		Body weight (g) on days																	
		0	3	7	10	14	17	21	24	28	35	42	49	56	63	70	77	84	89
Control	Mean	237.4	249.8	273.7	280.1	302.4	309.9	329.5	334.7	351.7	371.9	388.3	403.0	416.2	425.4	432.7	443.2	446.0	451.6
	SD	12.7	15.6	18.2	21.0	23.7	22.5	25.5	25.9	27.0	28.1	31.4	31.4	33.2	37.4	35.2	37.9	35.2	37.9

500	Mean	240.7	255.3	281.0	288.5	311.6	315.5	335.9	343.7	358.0	376.4	390.6	404.6	418.6	427.9	437.8	444.2	445.5	451.1
	SD	12.2	12.6	14.6	14.7	16.7	17.2	18.1	18.8	20.4	19.8	19.1	21.5	21.4	23.4	25.3	22.0	23.3	23.5

1000	Mean	234.0	252.1	276.3	284.7	305.4	312.8	329.1	336.3	346.9	367.2	381.0	393.9	405.7	417.0	423.5	429.0	431.2	438.8
	SD	11.1	14.8	20.0	21.1	24.5	27.0	31.1	32.6	35.6	39.0	41.6	43.2	42.9	48.2	48.8	51.1	53.9	54.4

2000	Mean	234.0	249.9	272.3	279.2	298.8	300.8	318.5	323.0	333.3	350.2	360.4	369.0	379.9	389.9	401.5	407.2	412.8	410.8
	SD	10.3	12.1	14.8	16.3	19.2	18.6	21.8	23.6	24.8	28.6	32.3	37.0	38.6	35.1	30.3	28.2	30.1	30.5
	SS												^*∗*^	^*∗*^					^*∗*^

Test for significance		NS	NS	NS	NS	NS	NS	NS	NS	NS	NS	NS	DN	DN	NS	NS	NS	NS	DN

Abbreviations: DN: Duncan's multiple range test; NS: not significant; SD: standard deviation; SS: statistical significance. ^‡^*n* = 10 for all groups. ^*∗*^=*p* < 0.05.

**Table 6:** Summary of body weight gain between days (males), 90-day study.

Group^‡^ mg/kg bw/day		Body weight gain (g) between days																	Sum.
		0–3	3–7	7–10	10–14	14–17	17–21	21–24	24–28	28–35	35–42	42–49	49–56	56–63	63–70	70–77	77–84	84–89	0–89
Control	Mean	12.4	23.9	6.4	22.3	7.5	19.6	5.2	17.0	20.2	16.4	14.7	13.2	9.2	7.3	10.5	2.8	5.6	214.2
	SD	4.0	3.6	5.2	3.4	4.1	3.6	2.0	2.7	3.5	4.8	4.4	3.9	5.3	3.9	5.0	4.0	4.5	28.5

500	Mean	14.6	25.7	7.5	23.1	3.9	20.4	7.8	14.3	18.4	14.2	14.0	14.0	9.3	9.9	6.4	1.3	5.6	210.4
	SD	3.1	3.0	3.4	3.5	4.6	3.5	2.2	3.7	4.1	3.0	4.1	4.8	4.7	3.1	5.6	4.3	4.2	16.3
	SS							^*∗*^											

1000	Mean	18.1	24.2	8.4	20.7	7.4	16.3	7.2	10.6	20.3	13.8	12.9	11.8	11.3	6.5	5.5	2.2	7.6	204.8
	SD	4.6	7.1	2.8	4.9	3.7	5.5	2.8	4.5	4.9	5.8	4.0	2.9	6.8	4.4	4.7	6.1	3.9	47.7
	SS	^*∗∗*^							^*∗∗*^							^*∗*^			

2000	Mean	15.9	22.4	6.9	19.6	2.0	17.7	4.5	10.3	16.9	10.2	8.6	10.9	10.0	11.6	5.7	5.6	-2.0	176.8
	SD	3.4	3.7	2.6	4.8	4.3	3.9	3.2	4.5	5.8	5.8	7.7	4.2	9.4	9.2	12.6	6.1	3.7	23.4
	SS					^*∗*^			^*∗∗*^		^*∗*^	^*∗*^				^*∗*^		^*∗∗*^	^*∗∗*^

Test for significance		DN	NS	NS	NS	DN	NS	DN	DN	NS	DN	DN	NS	NS	NS	*U*	NS	DN	*U*

Abbreviations: DN: Duncan's multiple range test; NS: not significant; SD: standard deviation; SS: statistical significance; U: Mann–Whitney U-test versus control. ^‡^*n* = 10 for all groups. ^*∗*^*p* < 0.05, ^*∗∗*^*p* < 0.01.

**Table 7:** Summary of feed efficiency (males), 90-day study.

(Group mg/kg bw/day)		Feed efficiency (g food/g bwg)													
		Days													
		0–7	7–14	14–21	21–28	28–35	35–42	42–49	49–56	56–63	63–70	70–77	77–84	84–89	0–89
		Weeks													
		1	2	3	4	5	6	7	8	9	10	11	12	13	1–13
Control	Mean	4.84	5.94	6.44	7.43	8.20	10.68	12.05	13.31	30.53	22.14	19.73	49.20	32.23	9.94
	SD	0.61	1.03	1.32	0.76	1.43	2.48	3.59	3.43	45.68	11.08	8.06	53.83	35.21	0.79
	*n* ^†^	10	10	10	10	10	10	10	10	9	9	10	6	8	10

500	Mean	4.47	5.71	7.44	7.92	9.38	12.43	13.32	13.09	21.55	18.41	26.83	73.06	34.05	10.32
	SD	0.42	0.64	1.90	1.89	2.39	2.72	5.41	3.78	7.57	7.15	15.00	65.90	35.36	0.65
	*n* ^†^	10	10	10	10	10	10	10	10	10	10	8	6	9	10

1000	Mean	4.17	5.95	7.65	9.68	8.29	13.33	14.08	14.73	22.40	28.31	26.18	34.60	16.41	10.49
	SD	0.89	1.14	1.97	2.01	1.49	4.12	6.45	4.19	18.82	15.81	9.75	22.24	5.85	1.53
	*n* ^†^	10	10	10	10	10	10	10	10	10	9	8	6	9	10
	SS				^*∗*^										

2000	Mean	4.49	6.55	8.86	12.91	10.20	16.41	16.78	19.38	37.31	17.86	66.44	39.20	76.28	11.82
	SD	0.54	1.19	1.54	7.33	3.09	7.17	6.07	14.54	42.43	7.30	72.73	45.20	41.40	1.32
	*n* ^†^	10	10	10	10	10	9	9	10	10	10	7	9	3	10
	SS			^*∗∗*^	^*∗∗*^									^*∗*^	^*∗∗*^

Test for significance		NS	NS	DN	U	NS	NS	NS	NS	NS	NS	NS	NS	U	DN

Abbreviations: DN: Duncan's multiple range test; NS: not significant; SD: standard deviation; SS: statistical significance; U: Mann–Whitney U-test versus control. ^†^Group “*n*”s were reduced for some weeks due to individual animals either having (0) weight gain or weight loss. ^*∗*^*p* < 0.05, ^*∗∗*^*p* < 0.01.

**Table 8:** Summary of relevant^a^ hematology results, 90-day study.

Group^‡^ mg/kg bw/day		EOS (%)	MCV (fL)	PLT (×10^9^/L)	RET (%)
*Males*					
Control	Mean	2.07	54.28	679.0	1.80
	SD	0.56	2.32	150.0	0.20
500	Mean	2.48	52.44	739.1	1.60
	SD	3.12	1.50	154.1	0.15
	SS		^*∗*^		
1000	Mean	1.79	53.98	741.3	1.54
	SD	1.02	1.51	90.4	0.24
	SS				^*∗*^
2000	Mean	1.21	53.02	818.4	1.55
	SD	0.28	1.72	111.4	0.34
	SS	^*∗∗*^			^*∗*^
Test for significance		U	DN	NS	DN
Historical control range		0.3–9.0	45.4–53.7	595–957	2.05–4.65

*Females*					
Control	Mean	2.23	55.63	707.2	2.18
	SD	0.84	1.56	151.8	0.34
500	Mean	1.64	55.32	786.3	1.93
	SD	0.81	1.36	136.1	0.36
1000	Mean	1.49	53.97	892.6	1.80
	SD	0.37	1.43	100.1	0.40
	SS			^*∗∗*^	
2000	Mean	1.52	54.69	849.4	1.90
	SD	0.81	1.97	109.3	0.34
	SS			^*∗*^	
Test for significance		NS	NS	DN	NS
Historical control range		0.4–2.1	47.0–60.1	549–1103	2.77–5.63

Abbreviations: EOS: eosinophils; DN: Duncan's multiple range test; MCV: mean corpuscular volume; NS: nonsignificant; PLT: platelets; RET: reticulocytes; SD: standard deviation; SS: statistical significance; U: Mann–Whitney U-test versus control. ^a^ Only statistically significant findings are shown. ^‡^*n* = 10 for all groups. ^*∗*^*p* < 0.05, ^*∗∗*^*p* < 0.01.

**Table 9:** Summary of clinical chemistry results, 90-day study.

Group^‡^ mg/kg bw/day		ALT (U/L)	AST (U/L)	ALP (U/L)	TBIL (*μ*mol/L)	CREA (*μ*mol/L)	Urea (mmol/L)	GLUC (mmol/L)	CHOL (mmol/L)	Pi (mmol/L)	Ca^++^ (mmol/L)	Na^+^ (mmol/L)	K^+^ (mmol/L)	Cl^−^ (mmol/L)	ALB (g/L)	TPROT (g/L)	A/G
*Males*																	
Control	Mean	51.5	86.4	142.0	1.02	25.4	7.90	7.56	2.35	1.91	2.68	142.94	4.59	98.85	44.29	65.91	2.08
	SD	8.8	11.0	41.2	0.41	2.5	0.51	0.89	0.41	0.13	0.06	0.97	0.33	1.48	1.78	2.24	0.30
500	Mean	49.5	80.3	125.4	1.00	25.7	7.21	7.35	2.56	1.90	2.66	142.28	4.35	98.55	43.54	64.80	2.06
	SD	6.7	10.0	27.7	0.32	2.5	0.51	0.34	0.40	0.19	0.05	0.50	0.23	0.78	1.37	2.30	0.19
1000	Mean	54.6	84.6	107.1	0.85	26.4	7.18	7.47	2.37	1.98	2.65	142.28	4.33	98.13	43.54	63.74	2.16
	SD	9.5	18.1	24.7	0.35	3.5	0.92	0.67	0.38	0.17	0.06	1.49	0.31	2.00	1.10	2.09	0.16
	SS			^*∗*^												^*∗*^	
2000	Mean	46.0	79.9	110.1	1.18	24.5	6.93	6.85	2.09	1.87	2.64	142.37	4.50	97.36	42.63	60.98	2.34
	SD	5.2	12.6	21.7	0.36	2.3	1.06	0.37	0.26	0.21	0.05	0.68	0.30	0.99	0.74	1.12	0.19
	SS			^*∗*^			^*∗*^							^*∗*^	^*∗*^	^*∗∗*^	^*∗*^
Test for significance		NS	NS	DN	NS	NS	DN	NS	NS	NS	NS	NS	NS	U	U	DN	DN
Historical control range		28.0–86.2	67.9–135.7	56–184	0.71–2.79	18.5–37.2	3.60–9.26	4.58–8.24	1.27–2.44	1.40–2.41	2.40–2.80	140–146	3.84–5.04	102.7–106.9	33.0–37.4	56.8–68.0	1.0–1.4

*Females*																	
Control	Mean	86.1	141.4	65.6	1.46	31.20	6.91	6.54	2.11	1.18	2.67	143.50	4.04	101.44	50.88	69.11	2.80
	SD	32.1	51.1	38.0	0.48	2.49	1.26	0.62	0.32	0.30	0.09	2.01	0.37	2.22	2.50	2.98	0.22
500	Mean	59.9	101.0	62.7	1.12	29.30	6.83	7.11	1.81	1.36	2.65	142.72	4.09	100.38	50.42	67.53	2.96
	SD	16.9	13.6	16.1	0.20	3.71	0.87	0.42	0.40	0.27	0.08	1.49	0.39	1.67	2.69	3.42	0.30
	SS	^*∗*^						^*∗*^									
1000	Mean	73.5	106.3	48.7	1.34	27.00	7.08	6.59	2.10	1.66	2.67	140.93	3.99	98.41	49.77	67.47	2.85
	SD	22.2	22.2	11.7	0.36	2.62	1.16	0.56	0.38	0.31	0.11	1.14	0.21	1.85	3.20	3.84	0.40
	SS					^*∗∗*^				^*∗∗*^		^*∗∗*^		^*∗∗*^			
2000	Mean	61.3	97.1	58.2	1.08	27.20	6.90	6.78	2.08	1.71	2.67	140.92	3.95	97.43	47.83	64.62	2.87
	SD	20.1	20.4	26.6	0.36	2.90	1.12	0.51	0.34	0.27	0.05	1.42	0.11	1.27	2.23	1.76	0.41
	SS				^*∗*^	^*∗∗*^				^*∗∗*^		^*∗∗*^		^*∗∗*^	^*∗*^	^*∗∗*^	
Test for significance		DN	NS	NS	DN	DN	NS	DN	NS	DN	NS	DN	NS	DN	DN	DN	NS
Historical control range		23.4–87.7	71.0–141.8	25.0–126	1.23–3.30	25.6–40.8	4.25–8.5	4.44–7.55	1.35–3.39	0.93–2.01	2.51–2.77	135–148	3.42–4.35	103.3–109.6	33.2–41.0	57.1–74.5	1.1–1.5

Abbreviations: A/G: albumin/globulin; ALP: alkaline phosphatase; ALB: albumin; ALT: alanine aminotransferase; AST: aspartate aminotransferase; Ca^++^: calcium; CHOL: cholesterol; Cl^−^: chloride; CREA: creatinine; DN: Duncan's multiple range test; GLUC: glucose; Na+: sodium; NS: not significant; Pi: inorganic phosphorus; SD: standard deviation; SS: statistical significance; U: Mann–Whitney U-test versus control; TBIL: total bilirubin; TPROT: total protein; ^‡^*n* = 10 for all groups. ^*∗*^*p* < 0.05, ^*∗∗*^*p* < 0.01.

**Table 10:** Summary of macroscopic results, 90-day study.

Organs	Group^‡^ mg/kg bw/day	Control	500	1000	2000
	Observations	(# with observation/# observed)			
Males	No macroscopic findings	8/10	6/10	9/10	5/10
Thymus	Hemorrhage	0/10	1/10	0/10	0/10
Liver	Congestion	0/10	0/10	0/10	1/10
Kidneys	Cyst	1/10	0/10	0/10	0/10
	Pyelectasia	0/10	1/10	1/10	2/10
Epididymides	Yellow knots on the tail	0/10	1/10	0/10	0/10
Spleen	Formation on the surface	0/10	0/10	0/10	1/10
Cecum	Dilatation	0/10	0/10	0/10	1/10
Skin	Alopecia	1/10	1/10	0/10	0/10

Females	No macroscopic findings	2/10	7/10	5/10	8/10
Lungs	Point-like hemorrhages	1/10	0/10	0/10	0/10
Kidneys	Pyelectasia	2/10	1/10	0/10	0/10
	Hydronephrosis	1/10	0/10	0/10	0/10
Ureter	Dilatation	1/10	0/10	0/10	0/10
Thymus	Hemorrhages	1/10	0/10	1/10	2/10
Uterus	Hydrometra	6/10	3/10	5/10	0/10
Skin	Alopecia	1/10	0/10	0/10	0/10

^‡^ *n* = 10 for all groups.

**Table 11:** Summary of relevant^a^ absolute and relative organ weights.

Organ weight (g)					Organ weight relative to body weight (%)						Organ weight relative to brain and body weights (%)
Group^‡^ mg/kg bw/day		Body weight	Heart	Testes/uterus	Brain	Liver	Kidneys	Heart	Testes/uterus	Adrenals	Liver
*Males*											
Control	Mean	453.90	1.03	3.69	0.488	2.918	0.558	0.228	0.813	0.013	599.45
	SD	37.20	0.10	0.36	0.033	0.177	0.041	0.015	0.061	0.002	31.07
500	Mean	449.4	1.07	3.60	0.486	2.872	0.557	0.239	0.800	0.014	591.18
	SD	26.28	0.06	0.25	0.022	0.215	0.026	0.013	0.039	0.002	37.77
1000	Mean	435.8	1.07	3.53	0.491	2.883	0.576	0.245	0.813	0.014	591.36
	SD	52.01	0.13	0.47	0.047	0.255	0.036	0.008	0.083	0.003	73.56
	SS							^*∗∗*^			
2000	Mean	408.3	0.93	3.60	0.521	2.984	0.609	0.229	0.884	0.016	574.29
	SD	29.98	0.08	0.21	0.034	0.255	0.055	0.015	0.062	0.003	59.66
	SS	^*∗*^	^*∗*^		^*∗*^		^*∗*^		^*∗*^	^*∗*^	
Test for significance		DN	DN	NS	DN	NS	DN	DN	DN	DN	NS
Historical control range		344–488	0.97–1.50	2.58–4.20	0.441–0.599	1.916–3.108	0.466–0.650	0.216–0.311	0.642–1.011	0.012–0.023	371.50–660.20

*Females*											
Control	Mean	239.3	0.72	0.77	0.825	2.659	0.659	0.301	0.322	0.0331	324.64
	SD	15.83	0.07	0.16	0.066	0.148	0.106	0.022	0.073	0.0042	34.68
500	Mean	242.0	0.70	0.67	0.812	2.881	0.642	0.290	0.281	0.0345	357.26
	SD	16.19	0.08	0.14	0.070	0.318	0.065	0.030	0.071	0.0046	48.82
	SS					^*∗*^					
1000	Mean	234.3	0.68	0.67	0.826	2.749	0.648	0.289	0.287	0.0328	333.21
	SD	11.29	0.03	0.16	0.048	0.173	0.030	0.018	0.075	0.0043	22.17
2000	Mean	236.8	0.69	0.57	0.803	2.883	0.660	0.290	0.243	0.0321	360.17
	SD	17.61	0.06	0.11	0.060	0.176	0.046	0.018	0.050	0.0027	28.19
	SS			^*∗∗*^		^*∗*^			^*∗*^		^*∗*^
Test for significance		NS	NS	DN	NS	DN	NS	NS	DN	NS	DN
Historical control range		206–285	0.66–0.96	0.40–2.07	0.681–0.943	2.172–3.214	0.530–0.752	0.273–0.396	0.167–0.852	0.026–0.044	276.04–412.95

Abbreviations: DN: Duncan's multiple range test; SD: standard deviation; SS: statistical significance; NS: nonsignificant; ^a^ Only statistically significant findings are shown. ^‡^*n* = 10 for all groups. ^*∗*^*p* < 0.05, ^*∗∗*^*p* < 0.01.

**Table 12:** Summary of histopathology, 90-day study.

Organs	Group^‡^ mg/kg bw/day	Control	500	1000	2000
	Observations	(# with observation/# observed)			
Males	Animals without microscopic findings	9/10	N/A	N/A	4/10
Cecum	Dilatation	0/10	—	—	1/10
Epididymides	Sperm granuloma	0/10	1/1	—	0/10
Kidneys	Pyelectasia	0/10	1/1	1/1	2/10
	Cyst	1/10	0/1	0/1	0/10
Liver	Congestion	0/10	—	—	1/10
Lungs	Alveolar emphysema	1/10	—	—	1/10
	Hyperplasia of BALT	1/10	—	—	0/10
Skin	Atrophy of hair follicles	0/10	1/1	—	0/10
Spleen	Hyperplasia	0/10	—	—	1/10
Thymus	Acute hemorrhage	0/10	1/1	—	0/10

Females	Animals without microscopic findings	6/10	N/A	N/A	8/10
Kidneys	Pyelectasia	0/10	1/1	—	0/10
	Hydronephrosis	1/10	0/1	—	0/10
Lungs	Alveolar emphysema	1/10	—	—	0/10
	Acute hemorrhage	1/10	—	—	0/10
Skin	Atrophy of hair follicles (focal)	1/10	—	—	0/10
Thymus	Acute hemorrhage	1/10	—	1/1	2/10
Uterus	Dilatation	6/10	—	1/1	0/10

Abbreviations: BALT: bronchus-associated lymphoid tissue; N/A: not applicable. ^‡^*n* = 10 for all groups.

## Data Availability

The mean data sets generated and utilized for statistical analysis to support the findings of these studies are included within the article or in the supplementary information files. All other raw and processed data used to support the findings of these studies are available from the corresponding author upon request. All experimental records, specimens, and data are archived in compliance with GLP in the archives of TOXI-COOP, Zrt. in Balantonfüred, Hungary.

## Abbreviations

2AA: 2-aminoanthracene
9AA: 9-aminoacridine
bw: Body weight
DME: Dulbecco's modified Eagle's
DMSO: Dimethyl sulfoxide
EMS: Ethyl methanesulfonate
EPA: Environmental protection agency
FOB: Functional observation battery
GLP: Good laboratory practices
MPCE: Micronucleated polychromatic erythrocytes
MMS: Methyl-methanesulfonate
NOAEL: No-observed-adverse-effect level
NPD: 4-Nitro-1,2-phenylene-diamine
OECD: Organization of economic cooperation and development
OPPTS: Office of prevention, pesticides, and toxic substances
PCE: Polychromatic erythrocytes
SAZ: Sodium azide.

## Supplementary Materials

Supplementary Materials Complete summary data tables for the 90-day repeated-dose oral toxicity study are available in Supplementary Material for the following parameters (reported as means ± standard deviations): body weights (female), body weight gain (female), food consumption (males and females), food efficiency (female), hematology (males and females), and absolute and relative (to brain and body weight) organ weights (males and females).
